# Triple-zero tillage and system intensification lead to enhanced productivity, micronutrient biofortification and moisture-stress tolerance ability in chickpea in a pearlmillet-chickpea cropping system of semi-arid climate

**DOI:** 10.1038/s41598-023-36044-0

**Published:** 2023-06-23

**Authors:** Ram Swaroop Bana, Mukhtar Ahmad Faiz, Seema Sangwan, Anil K. Choudhary, Shanti D. Bamboriya, Samarth Godara, Ravi Chandrabhan Nirmal

**Affiliations:** 1 ICAR-Indian Agricultural Research Institute, New Delhi, 110012 India; 2 Afghanistan National Agricultural Sciences and Technology University (ANASTU), Kandahar, Afghanistan; 3 ICAR-Central Potato Research Institute, Shimla, Himachal Pradesh 171001 India; 4 ICAR-Indian Institute of Maize Research, Ludhiana, Punjab 141004 India; 5 ICAR-Indian Agricultural Statistics Research Institute, New Delhi, 110012 India

**Keywords:** Biochemistry, Microbiology, Plant sciences, Environmental sciences

## Abstract

Pearlmillet-chickpea cropping system (PCCS) is emerging as an important sequence in semi-arid regions of south-Asia owing to less water-requirement. However, chickpea (dry-season crop) faces comparatively acute soil moisture-deficit over pearlmillet (wet-season crop), limiting overall sustainability of PCCS. Hence, moisture-management (specifically in chickpea) and system intensification is highly essential for sustaining the PCCS in holistic manner. Since, conservation agriculture (CA) has emerged is an important climate-smart strategy to combat moisture-stress alongwith other production-vulnerabilities. Hence, current study comprised of three tillage systems in main-plots viz., Complete-CA with residue retention (CA_c_), Partial-CA without residue-retention (CA_p_), and Conventional-tillage (ConvTill) under three cropping systems in sub-plots viz., conventionally grown pearlmillet-chickpea cropping system (PCCS) alongwith two intensified systems i.e. pearlmillet-chickpea-fodder pearlmillet cropping system (PCFCS) and pearlmillet-chickpea-mungbean cropping system (PCMCS) in split-plot design. The investigation outcomes mainly focused on chickpea (dry-season crop) revealed that, on an average, there was a significant increase in chickpea grain yield under CA_c_ to the tune of 27, 23.5 and 28.5% under PCCS, PCFCS and PCMCS, respectively over ConvTill. NPK uptake and micronutrient (Fe and Zn) biofortification in chickpea grains were again significantly higher under triple zero-tilled CA_c_ plots with residue-retention; which was followed by triple zero-tilled CA_p_ plots without residue-retention and the ConvTill plots. Likewise, CA_c_ under PCMCS led to an increase in relative leaf water (RLW) content in chickpea by ~ 20.8% over ConvTill under PCCS, hence, ameliorating the moisture-stress effects. Interestingly, CA-management and system-intensification significantly enhanced the plant biochemical properties in chickpea viz*.,* super-oxide dismutase, ascorbate peroxidase, catalase and glutathione reductase; thus, indicating their prime role in inducing moisture-stress tolerance ability in moisture-starved chickpea. Triple zero-tilled CA_c_ plots also reduced the N_2_O fluxes in chickpea but with slightly higher CO_2_ emissions, however, curtailed the net GHG-emissions. Triple zero-tilled cropping systems (PCFCS and PCMCS) both under CA_c_ and Ca_p_ led to a significant improvement in soil microbial population and soil enzymes activities (alkaline phosphatase, fluorescein diacetate, dehydrogenase). Overall, the PCCS system-intensification with mungbean (PCMCS) alongwith triple zero-tillage with residue-retention (CA_c_) may amply enhance the productivity, micronutrient biofortification and moisture-stress tolerance ability in chickpea besides propelling the ecological benefits under semi-arid agro-ecologies. However, the farmers should preserve a balance while adopting CA_c_ or CA_p_ where livestock equally competes for quality fodder.

Pearlmillet-chickpea cropping system (PCCS) is an important crop sequences of semi-arid regions of south-Asia owing to less water-requirement^1^. In arid and semi-arid regions of south-Asia, pearlmillet (*Pennisetum glaucum* L.) is the main food crop grown in wet-season in mono- or double-cropping systems because of its hardy nature against extreme weather conditions^2^. Likewise, chickpea (*Cicer arietinum* L.), an important dry-season (*Rabi*) legume crop grown on residual soil moisture, immensely contributes towards soil fertility restoration and nutritional security in this region^1,3,4^. However, chickpea (dry-season crop) faces comparatively acute soil moisture-deficit in dry season due to erratic precipitation distribution over pearlmillet (wet-season crop), hence, limiting chickpea productivity and quality. These production-vulnerabilities pose a great threat to the sustainability of pearlmillet-chickpea cropping system (PCCS) in the region. Therefore, appropriate soil and moisture conservation practices can mitigate the aforesaid challenges in PCCS under such vulnerable ecologies.

Rainfed agriculture already contributes ~ 82% of global arable area (1.223 billion ha) while hosting ~ 40% global population^5^. Increasing population in south-Asia further exerts an exorbitant pressure to enhance the food production in arid and semi-arid regions. With the advent of short-duration pearlmillet varieties, it has though become possible to go in for triple cropping systems with appropriate soil and moisture management practices. Here, mungbean and fodder pearlmillet can be the appropriate short-duration summer season crops for the intensification of PCCS in semi-arid regions to meet both pulse and fodder requirement, respectively^6^. Hence, appropriate moisture-management (specifically in chickpea) coupled with system intensification may play an important role in sustaining the PCCS in holistic manner. Conservation agriculture (CA) has emerged as an important climate-smart strategy to mitigate the moisture stress in vulnerable agro-ecologies besides its positive impact on crop productivity, quality, moisture-stress tolerance and soil health^7–9^.

Residue retention on the soil surface under CA-management prevents the soil erosion and evaporation losses, and regulates the soil thermal dynamics which maintains soil moisture, and consequently improves water- and nutrient-use efficiency and crop productivity^10^. CA systems also curtail the nutrient (~ 30%), labour (~ 50%), and fuel (~ 65%) requirement in various crops^11–14^. CA management also promotes the soil organic carbon (SOC) storage, aggregate stability, and soil biological activity over intensive tillage^1,15–17^. In addition, improved soil physico-chemical and biological properties and micro-climate modulation under CA-management^9,10,18,19^, may also prove helpful in inducing the moisture-stress tolerance ability in rainfed crops^20^. Hence, it is obligatory to generate robust scientific evidences for CA based agronomic interventions for the water-scarce semi-arid regions that too under triple-zero tilled cropping systems, as most of the research till date is largely confined to irrigation-based rice–wheat cropping system (RWCS) in south-Asia^21^. As, *Rabi* season chickpea is more vulnerable to moisture-stress due to meager and erratic rainfall distribution^5^. Hence, we studied the impact of CA practices and the system intensification on the productivity, quality and moisture-stress tolerance ability of moisture-starved chickpea as an indicator crop under intensified PCCS. Current experimentation was therefore based on the hypothesis that the triple zero-till based system intensification of PCCS would lead to enhanced productivity, micronutrient biofortification and plant biochemical properties with reduced green-house gas (GHG) emissions under chickpea besides improved soil microbial population and soil enzymatic activities in semi-arid agro-ecologies of south-Asia.

## Results and discussion

### Soil microbial population

**Figure 1:**
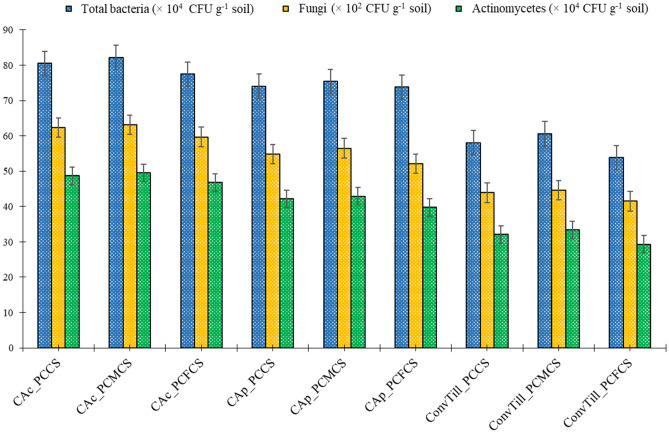
Effect of tillage practices and cropping systems on soil microbial populations (Soil samples taken from 0 to 15 cm soil layer after 2nd years of cropping cycle). Vertical bars indicate the LSD at* p* = 0.05. [*Note*: PCCS = Pearlmillet-chickpea cropping system; PCFCS = Pearl millet–chickpea–fodder pearlmillet cropping system; PCMCS = Pearlmillet-chickpea–mungbean cropping system; CA_c_ = Complete conservation agriculture with residue retention; CA_p_ = Partial conservation agriculture without residues; ConvTill = Conventional tillage].

In current study, soil microbial population was significantly (*p* < 0.05) affected by various tillage practices and the intensified cropping systems. The colony forming unit (CFU) counts were significantly higher under complete conservation agriculture (CA) with residue retention (CA_c_) followed by partial CA without residue retention (CA_p_) treatments and conventional tillage (ConvTill), respectively (Fig. 1). Highest number of bacteria, fungi and actinomycete were seen under CA_c_ followed by CA_p_. Across the tillage practices, pearlmillet-chickpea–mungbean cropping system (PCMCS) had significantly higher microbial CFU count followed by pearlmillet-chickpea cropping system (PCCS) while pearlmillet-chickpea–fodder pearlmillet cropping system (PCFCS) reported least microbial population. Among various treatment combinations, highest microbial counts of bacteria (82.2 × 10^4^ CFU g^−1^ soil), fungi (63.2 × 10^2^ CFU g^−1^ soil) and actinomycetes (49.5 × 10^4^ CFU g^−1^ soil) were observed in CA_c_ plots with PCMCS system (CA_c__PCMCS) followed by CA_c_ with PCCS system (CA_c__PCCS) and CA_c_ with PCFCS system (CA_c__PCFCS), respectively. Lowest population of bacteria (53.3 × 10^4^ CFU g^−1^ soil), fungi (41.5 × 10^2^ CFU g^−1^ soil) and actinomycetes (29.3 × 10^4^ CFU g^−1^ soil) were perceived under ConvTill_PCFCS which were 35.2, 52.2 and 16.2% lower than the best treatment combination CA_c__PCMCS. This may be accrued to the reason the high organic biomass addition under CA_c_ plots improved the soil structure, aggregate stability and uniform soil moisture availability, which in turn might have allowed microbial populations to grow and sustain in the rhizosphere^15–18,22^. Combining the CA_c_ practice with legume-intensification also enhanced the SOC input owing to adequate leaf litterfall and root biomass additions from legumes with narrow C:N ratio^18,19^; which in turn, enhanced the soil microbial diversity^17,23–25^. Legume roots also release the root exudates which harbor the microbial diversity in the rhizosphere^4,20,26^, that’s why the double-legumes system i.e. PCMCS had highest microbial counts of bacteria, fungi and actenomycetes in current study.

### Soil microbial enzymatic activities

**Figure 2:**
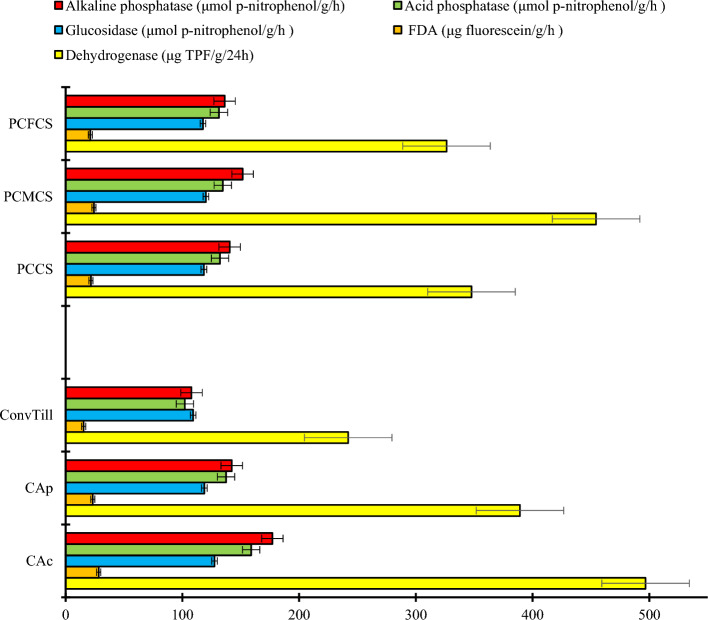
Effect of tillage practices and cropping systems on soil microbial enzyme activities (Soil samples taken from 0 to 15 cm soil layer after 2nd years of cropping cycle). Vertical bars indicate the LSD at* p* = 0.05. [*Note*: PCCS = Pearlmillet-chickpea cropping system; PCFCS = Pearl millet–chickpea–fodder pearlmillet cropping system; PCMCS = Pearlmillet-chickpea–mungbean cropping system; CA_c_ = Complete conservation agriculture with residue retention; _CAp_ = Partial conservation agriculture without residues; ConvTill = Conventional tillage].

Different tillage practices had significant (*p* < 0.05) effect on acid and alkaline phosphatase, glucosidase, dehydrogenase and fluorescein diacetate (FDA) activities (Fig. 2). These enzymatic activities were significantly (*p* < 0.05) higher under CA_c_ followed by CA_p_, and ConvTill. Compared to ConvTill, the acid phosphatase, alkaline phosphatase, glucosidase, dehydrogenase and FDA activities were higher by 55.6, 64.3, 16.7, 105.3 and 83.8%, respectively under CA_c_. The system-intensification had significant effect on alkaline phosphatase, dehydrogenase and FDA activities. Highest activities of alkaline phosphatase (152 μmol p-nitrophenol g^−1^ h^−1^), dehydrogenase (454 μg TPF g^−1^ 24 h^−1^) and FDA (24.2 μg fluorescein g^−1^ h^−1^) were observed under PCMCS, whereas PCFCS had least activities of these enzymes. As, legume-inclusion enhances the SOM^4,20,26^, thus, resulting in higher soil enzyme activities under double-legume system PCMCS^21,27^. Likewise, higher SOC enrichment both under CA-based tillage systems and the legume-intensification might have enhanced the FDA activity in our study^16,21^. The CA practices and legume-intervention also enhanced the dehydrogenase activity due to higher microbial nutrient bioavailability in the rhizosphere^16,28,29^.

### Crop productivity

**Figure 3:**
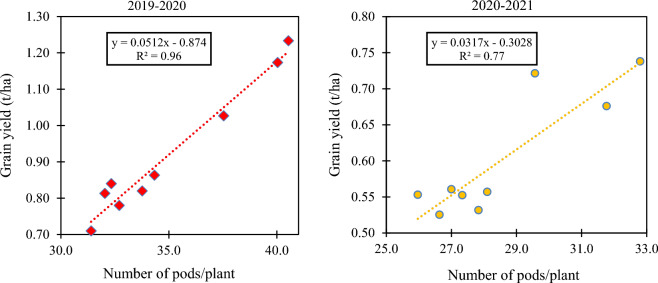
Correlation between chickpea grain yield and pods plant^-1^ under different cropping systems for 2-years, i.e., 2019–2020 and 2020–21.

Different Tillage practices and cropping systems had significant (*p* < 0.05) influence on the number of pods plant^−1^ during both years (Table 1). Pods plant^−1^ during 2020–2021 were 19% lesser than the year 2019–2020. Highest pods plant^−1^ (40.5 and 32.8) were obtained under CA_c__PCMCS compared to rest of the treatments combinations during both years where ConvTill_PCFCS had least pod count plant^−1^ (31.4 and 26.6). Crop residue-retention improves the soil fertility and moisture holding capacity owing to SOM enrichment and nutrient bioavailability after biomass decomposition^13,21,30^, which accelerate the plant growth and dry matter accumulation and finally economic yield^31^. The CA practices are intended to increase carbon inputs, nutrient bioavailability with better physical rhizo-ecology (aggregate formation, moisture permeability and conservation) which directly proliferate the soil microbial diversity with higher crop yields^13,16^. Similarly, the interaction tillage and legume-inclusion (mungbean) showed significant (*p* < 0.05) grain and straw yield enhancement in chickpea during both years (Table 1). In general, chickpea grain and straw yield was comparatively higher during 2019–2020 than 2020–2021 owing to uniform rainfall distribution during 2019–2020 compared to 2020–2021 (Fig. 6). Significantly highest grain (1.23; 0.74 t ha^−1^) and straw yield (3.6; 2.06 t ha^−1^) of chickpea were recorded from the combination of CA_c_ with PCMCS system over other combinations during 2019–2020 and 2020–2021, respectively. The CA_c_ practice compared to ConvTill had a respective average grain yield increase by ~ 27, 23.5 and 28.5% and average straw yield increase by ~ 48.5, 47.5 and 56% under PCCS, PCFCS and PCMCS in our study. Again, the conventionally tilled PCFCS system had least grain and stover yield over other cropping systems. As, residue retention under CA plots was highly effective in reducing the evaporation losses and conserving more soil moisture, thus, resulting in better crop growth and yield over ConvTill plots^13,14^. Moreover, chickpea is a deep-rooted crop, therefore, which efficiently utilized the conserved soil moisture under CA plots for realizing higher yields^4,32^. There existed a significant positive and strong correlation between chickpea productivity and pods plant^-1^ during 2019–2020 (R^2^ = 0.96) and 2020–2021 (R^2^ = 0.77) (Fig. 3). The overall improvement in chickpea yield under CA plots (CA_c_ and CA_p_) could be ascribed to pivotal role of crop residues in several physiological, biochemical, chemical and physical processes^15–17,23,24,33^.

**Table 1:** Interaction effect of tillage practices × cropping systems on pods plant^−1^, grain and straw yields of chickpea.

Treatments* (Tillage practices × cropping systems)	PCCS		PCFCS		PCMCS	
	2019–2020	2020–2021	2019–2020	2020–2021	2019–2020	2020–2021
Pods plant^−1^						
CA_c_	40.0	29.5	37.5	31.7	40.5	32.8
CA_p_	33.8	27.3	32.7	27.8	34.3	28.1
ConvTill	32.0	25.9	31.4	26.6	32.5	27.0
LSD (*p* = 0.05) B at same level of A	2.5	1.8				
LSD (*p* = 0.05) A at same level of B	2.1	1.4				
Grain yield (t ha^−1^)						
CA_c_	1.17	0.72	1.02	0.68	1.23	0.74
CA_p_	0.82	0.55	0.78	0.53	0.86	0.56
ConvTill	0.81	0.54	0.71	0.52	0.84	0.56
LSD (*p* = 0.05) B at same level of A	0.04	0.03				
LSD (*p* = 0.05) A at same level of B	0.12	0.10				
Straw yield (t ha^−1^)						
CA_c_	3.25	2.00	3.01	1.85	3.60	2.06
CA_p_	2.81	1.52	2.60	1.43	3.04	1.58
ConvTill	2.82	1.48	2.48	1.41	2.98	1.56
LSD (*p* = 0.05) B at same level of A	0.08	0.06				
LSD (*p* = 0.05) A at same level of B	0.25	0.17				

*Note: PCCS = Pearlmillet-chickpea cropping system; PCFCS = Pearl millet–chickpea–fodder pearlmillet cropping system; PCMCS = Pearlmillet-chickpea–mungbean cropping system; CA_c_ = Complete conservation agriculture with residue retention; CA_p_ = Partial conservation agriculture without residues; ConvTill = Conventional tillage.

### Nutrient uptake

The experimental results revealed that both CA practices (CA_c_ and CA_p_) improved the total (grain + stover) NPK uptake in chickpea over conventional tillage (Table 2). Significantly (*p* < 0.05) higher total N (73.3 l; 43 kg ha^−1^), P (7.5; 4.3 kg ha^−1^) and K uptake (53.3; 30.2 kg ha^−1^) were obtained under CA_c__PCMCS during 2019–2020 and 2020–2021. Greater nutrient bioavailability as a result of optimal moisture conditions under CA plots could be the major factor for such observations^34^. Higher NPK uptake may also be accrued to higher yield under CA_c_ owing to improved soil physico-chemical and biological properties^9,10^. Lowest NPK uptake was recorded from ConvTill_PCFCS owing to poor crop growth and biomass production in ConvTill plots compared to CAc^18,19,35^. Higher NPK uptake under PCMCS may also be accrued to inclusion of two legumes (chickpea and mungbean) in the system which greatly improved the soil biofertility over the PCCS and PCFCS systems^4^.

**Table 2:** Interaction effect of tillage practices × cropping systems on total nutrient (N, P, K) uptake (kg ha^−1^) in chickpea.

Treatments* (Tillage practices × cropping systems)	PCCS		PCFCS		PCMCS	
	2019–2020	2020–2021	2019–2020	2020–2021	2019–2020	2020–2021
N uptake (kg ha^−1^)						
CA_c_	67.8	41.7	61.3	38.8	73.3	43.0
CA_p_	52.3	31.8	49.5	30.2	56.0	32.5
ConvTill	51.9	31.5	45.7	30.1	54.7	32.5
LSD (*p* = 0.05) B at same level of A	9.3	5.5				
LSD (*p* = 0.05) A at same level of B	12.4	8				
P uptake (kg ha^−1^)						
CA_c_	6.4	4.1	6.0	3.9	7.5	4.3
CA_p_	5.5	3.1	4.7	2.9	5.7	3.1
ConvTill	5.4	3.1	4.7	2.9	5.4	3.0
LSD (*p* = 0.05) B at same level of A	0.6	0.3				
LSD (*p* = 0.05) A at same level of B	0.75	0.6				
K uptake (kg ha^−1^)						
CA_c_	47.6	29.6	44.3	27.1	53.3	30.2
CA_p_	40.1	22.3	37.4	21.1	43.1	23.1
ConvTill	40.1	21.9	35.5	21	42.7	23.1
LSD (*p* = 0.05) B at same level of A	2.2	2.4				
LSD (*p* = 0.05) A at same level of B	5.3	5.7				

PCCS = Pearlmillet-chickpea cropping system; PCFCS = Pearl millet–chickpea–fodder pearlmillet cropping system; PCMCS = Pearlmillet-chickpea–mungbean cropping system; CA_c_ = Complete conservation agriculture with residue retention; CA_p_ = Partial conservation agriculture without residues; ConvTill = Conventional tillage.

### Micronutrient biofortification

Tillage practices and system-intensification had significant (*p* < 0.05) effect on micronutrient (Zn, Fe) biofortification in chickpea grains and straw (Table 3). Among tillage treatments, significantly (*p* < 0.05) greatest micronutrient content in chickpea grains as well as straw were obtained under CA_c_ and CA_p_ followed by ConvTill. The Fe and Zn content increased by ~ 2.5 and 1.56; and 8.3 and 10.1% in chickpea grains; and 3.4 and 3.8; and 3.7 and 6.2% in straw during 2019–2020 and 2020–2021, respectively over ConvTill. The improvement in micronutrient content under CA_c_ may be attributed to enhanced microbial activity and synchronous nutrient release during SOM decomposition process of the crop residues^16,24,36,37^. Likewise, the highest micronutrient content (Zn, Fe) in chickpea grains and straw were observed under PCMCS owing to higher nutrient acquisition and biomass productivity under the influence of two legumes i.e. chickpea and mungbean^4^. Significant enhancement in micronutrients (2-years’ av.) under different cropping systems was found to be 1.60 and 1.80 (Fe); 3.9 and 3.5 (Zn) mg kg^−1^ in grain and stover in PCCS and PCMCS, respectively over PCFCS. As, legume-imbedded systems fixed more N with sufficient biomass additions having narrow C: N ratio^18,19^; thus, speeding-up the biomass decomposition with more C-sequestration vis-à-vis more micronutrient acquisition^4,27^. The resultant SOM might have also helped in synthesis of organic acids in rhizosphere^27^, which in turn, acted as micronutrient chelates, influencing translocation and remobilization of micronutrients^37,38^.

**Table 3:** Effect of tillage practices and cropping systems on micronutrient (Fe, Zn) biofortification in chickpea grains.

Treatments	Grain				Straw			
	Fe (mg kg^−1^)		Zn (mg kg^−1^)		Fe (mg kg^−1^)		Zn (mg kg^−1^)	
	2019–2020	2020–2021	2019–2020	2020–2021	2019–2020	2020–2021	2019–2020	2020–2021
Tillage practices								
CA_c_	66.6	66.7	48.9	50.2	71.1	71.8	40.2	41.9
CA_p_	65.9	66.4	47.5	48.2	70.5	71.1	40.0	41.2
ConvTill	64.9	65.7	45.2	45.6	68.8	69.2	38.8	39.5
LSD (*p* = 0.05)	0.72	0.7	1.96	2.1	0.79	0.76	0.4	0.8
Cropping systems								
PCCS	65.8	66.3	47.2	48.4	70.2	70.7	39.7	40.8
PCFCS	65.3	65.7	46.0	46.6	69.5	69.9	39.1	40.6
PCMCS	66.3	66.7	48.4	49.0	70.8	71.4	40.1	41.3
LSD (*p* = 0.05)	0.54	0.53	1.3	1.4	0.57	0.61	0.5	0.6

PCCS = Pearlmillet-chickpea cropping system; PCFCS = Pearl millet–chickpea–fodder pearlmillet cropping system; PCMCS = Pearlmillet-chickpea–mungbean cropping system; CA_c_ = Complete conservation agriculture with residue retention; CA_p_ = Partial conservation agriculture without residues; ConvTill = Conventional tillage.

### Relative water content

**Figure 4:**
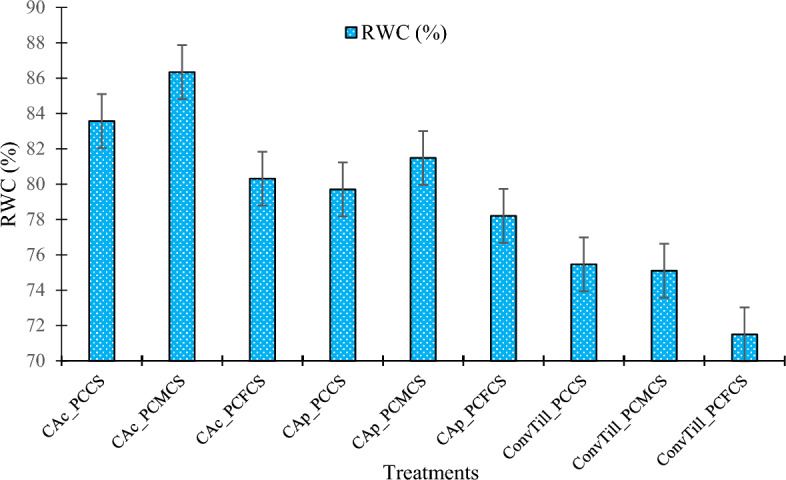
Effect of tillage practices and cropping systems on relative water content (RWC) in chickpea leaves (Pooled mean of 2-years). Vertical bars indicate the LSD at* p* = 0.05. [*Note*: PCCS = Pearlmillet-chickpea cropping system; PCFCS = Pearl millet–chickpea–fodder pearlmillet cropping system; PCMCS = Pearlmillet-chickpea–mungbean cropping system; CA_c_ = Complete conservation agriculture with residue retention; CA_p_ = Partial conservation agriculture without residues; ConvTill = Conventional tillage].

Various treatment combinations significantly (*p* < 0.05) improved the relative water content (RWC) in fully expanded chickpea leaves at flowering (Fig. 4). The highest RWC (86.3%) was achieved under CA_c_ in PCMCS system. This treatment combination improved the RWC by ~ 20.76% over ConvTill_PCFCS system. The improved RWC under CA_c_ was a consequence of higher moisture retention and comparatively lower moisture stress in residue-retained CA_c_ plots^9,10^. As, the legume intervention in the crop sequences enhances the water holding capacity due to better physical and biological rhizospheric environment, hence, resulting in favorable plant-soil–water relations with higher RWC^18,21,25^.

### Soil moisture content

**Figure 5:**
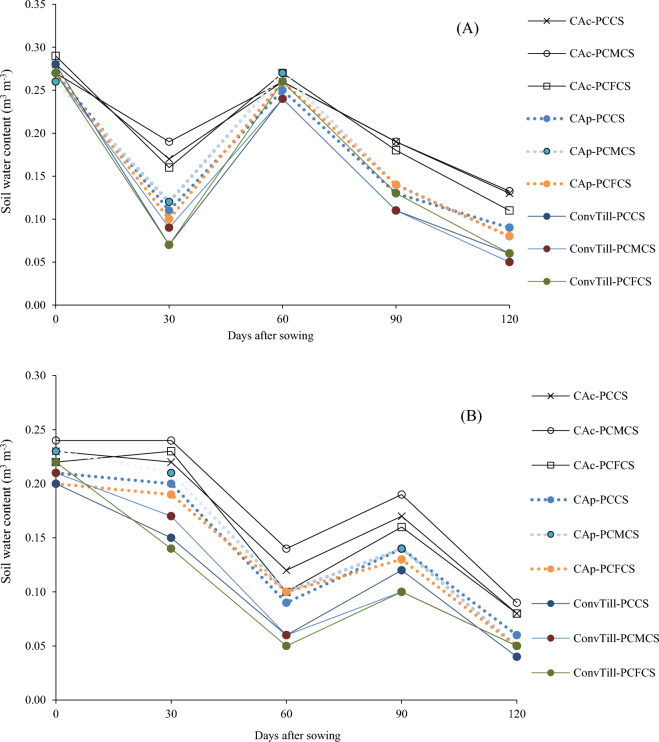
Soil moisture content at monthly interval during the chickpea crop growing period in the 0–15 cm soil profile corresponding to the year (**A**) 2019–20 and (**B**) 2020–21. [*Note*: PCCS = Pearlmillet-chickpea cropping system; PCFCS = Pearl millet–chickpea–fodder pearlmillet cropping system; PCMCS = Pearlmillet-chickpea–mungbean cropping system; CA_c_ = Complete conservation agriculture with residue retention; CA_p_ = Partial conservation agriculture without residues; ConvTill = Conventional tillage].

Diverse tillage and cropping system treatment combinations remarkably affected the soil moisture content (0–15 cm soil profile), at monthly intervals during both chickpea growing seasons (Fig. 5). The differences in soil moisture contents were relatively lesser during the crop period 2019–20 as the crop received 336.7 mm rainfall, in comparison to 2nd year when the winter season rainfall was merely 73.7 mm. Maximum soil moisture content were recorded under conservation agricultural systems, specifically under CAc plots. Whereas, under ConvTill treatments, the soil moisture remained lowest. The reduced moisture losses owing to lesser evaporation and greater moisture retention under crop residue retained plots might have resulted in more moisture availability under CA systems^2,10^. Likewise, the legume component in PCMCS under different tillage systems might have enhanced the water holding capacity due to better physical and biological rhizospheric environment, hence, resulting in greater moisture content in the soil profile^18,21^.

### Biochemical properties vis-à-vis moisture-stress tolerance ability

**Figure 6:**
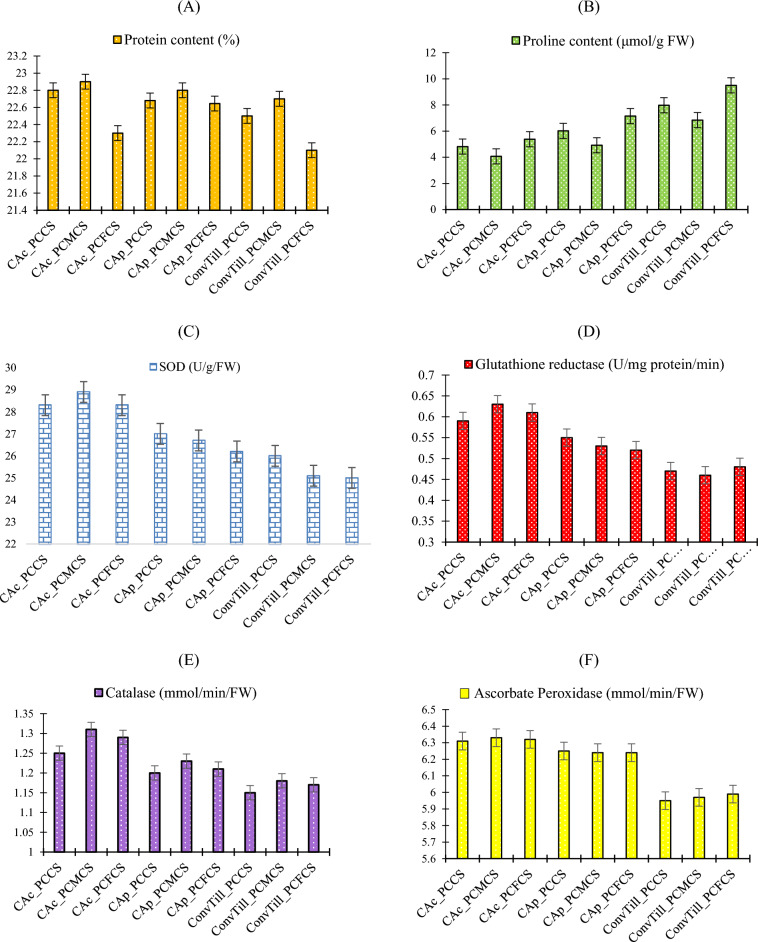
Effect of tillage practices and cropping systems on biochemical properties of chickpea (Pooled mean of 2-years), (**A**) Protein content; (**B**) Proline content; (**C**) Superoxide dismutase (SOD); (**D**) Ascorbate peroxidase (APX); (**E**) Catalase (CAT), and (**F**) Glutathione reductase (GR). Vertical bars indicate the LSD at* p* = 0.05. [*Note*: PCCS = Pearlmillet-chickpea cropping system; PCFCS = Pearl millet–chickpea–fodder pearlmillet cropping system; PCMCS = Pearlmillet-chickpea–mungbean cropping system; CA_c_ = Complete conservation agriculture with residue retention; CA_p_ = Partial conservation agriculture without residues; ConvTill = Conventional tillage; Vertical bars indicate the LSD at* p* = 0.05].

Tillage practices and system-intensification had significant (*p* < 0.05) influence on biochemical properties vis-à-vis moisture-stress tolerance ability of chickpea (Fig. 6), except ascorbate peroxidase (APX) and catalase (CAT) activity. Treatments, CA_c__PCMCS (22.9%), a combination of complete CA and double legume imbedded cropping system exhibited highest grain protein content (Fig. 6A), with ~ 3.6% higher protein content compared to ConvTill_PCCS. Higher N content in chickpea under CA_c__PCMCS may be attributed to increased N-bioavailability in the soil due to double legume-inclusion^1,4,18^. Higher decomposition rate of crop residues in CAc system might have also enhanced the N-acquisition and protein content in the plants^18,39^. Grain protein content was least in ConvTill_PCFCS be due to extensive N removal by two cereal components in the system^9,10^. The proline content was found to be inversely related with RWC. The maxima of proline content (9.5 μmol g^-1^ FW) was obtained under ConvTill_PCFCS (Fig. 6B). This treatment combination remained at par with ConvTill_PCCS (7.98 μmol g^−1^ FW) and ConvTill_PCMCS (6.84 μmol g^−1^ FW) and CAp_PCFCS (7.15 μmol g^−1^ FW). The least proline content was noticed in CAc_PCMCS and CAc_PCCS. The reduced proline levels in chickpea leaves in CA_c_ might be due to the increased moisture retention under crop residues, which resulted in low plant moisture-stress^25,40^. Similarly, chickpea plants grown with CA practices in PCMCS showed significantly higher biochemical properties like superoxide dismutase (SOD) activity (28.9 Ug^−1^ FW^−1^), and glutathione reductase (GR) activity (0.63 U mg^−1^ protein^−1^ min^−1^) which were ~ 11 and 30% higher over ConvTill_PCCS (Fig. 6C,D). Statistically non-significant increase was noticed in the CAT and APX activities under CA_c_ (Fig. 6E,F). Least proline and higher values of SOD, GR, CAT and APX activity in chickpea under CA_c_ indicate the ability of CA-management on moisture-stress tolerance in the current study^2,18^. It is evident from various studies that moisture or drought stress causes oxidative stress by decreasing stomatal conductivity in the plants which confines CO_2_ influx in to the leaves^40^. Hence, there is reduction in the leaf internal CO_2_, causing formation of reactive oxygen species (ROS) mainly in plant cell, mitochondria, chloroplasts and peroxisomes^41^. In our study, there was higher production of SOD, GR, CAT and APX activity in chickpea under CA_c_. As, higher ROS production induces deleterious impact on plant cells; the plant defense system becomes active against ROS^42^; and releases non-enzymatic antioxidants (proline) and antioxidant enzymes (like CAT, SOD) and ascorbate–glutathione (AsA–GSH) cycle enzymes (like GR and APX) for detoxification of ROS and plant cell protection^40–43^. It indicates that enhanced SOD, GR, CAT and APX activities under CA_c_ inducts drought-stress tolerance ability in chickpea plants in semi-arid environment.

### GHG-emissions

In current study, the CO_2_ and N_2_O emissions ranged between 1757 and 2246 kg ha^−1^ and 332–345 kg ha^−1^, respectively under various tillage treatments (Table 4). The CAc system emitted relatively larger amount of CO_2_ followed by CA_p_ and ConvTill. The CA_p_ and ConvTill remained statistically at par in terms of CO_2_ emissions. Contrary to CO_2_, the N_2_O emission was the larger under ConvTill and lowest in CA_c_ plots; however, net GHG-emissions were least under CA_c_ compared to ConvTill. Likewise, the system-intensification (PCMCS and PCFCS) led to slight enhancement in CO_2_ emissions both of which remained statistically at par. Intensive cropping (PCMCS/PCFCS) didn’t affect N_2_O emissions where all the cropping systems behaved statistically similar. Zero-tillage with residue retention in intensive cropping systems increased the availability of organic carbon that might have resulted in enhanced soil respiration and CO_2_ release^44,45^. Presence of residue-cover reduces the N_2_O emissions^46^, and therefore, the slightly lower N_2_O flux was observed in the CA-systems^47^.

**Table 4:** Effect of tillage practices and cropping systems on GHG-emission under pearlmillet-based cropping systems.

Treatment*	CO_2_ (kg ha^−1^)	N_2_O (g ha^−1^)
Tillage practices		
CA_c_	2242.6	332.6
CA_p_	1800.2	336.3
ConvTill	1757.4	345.9
LSD (*p* = 0.05)	167.2	8.3
Cropping systems		
PCCS	1602.2	294.5
PCFCS	1729.9	298.9
PCMCS	1741.2	306.7
LSD (*2.7* = 0.05)	53.3	NS

*Note: PCCS = Pearlmillet-chickpea cropping system; PCFCS = Pearl millet–chickpea–fodder pearlmillet cropping system; PCMCS = Pearlmillet-chickpea–mungbean cropping system; CA_c_ = Complete conservation agriculture with residue retention; CA_p_ = Partial conservation agriculture without residues; ConvTill = Conventional tillage.

## Conclusions

In current study, the triple zero-till based system-intensification coupled with residue retention (CA_c_) may enhance the chickpea grain yield by ~ 25% over conventional tillage (ConvTill) systems in the moisture-starved semi-arid ecologies. Likewise, the double legume bound triple cropping system i.e. pearlmillet-chickpea–mungbean cropping system (PCMCS) under CA_c_ significantly enhanced the relative leaf water content (~ 21%), total NPK uptake, protein content, micronutrient (Fe, Zn) biofortification, soil microbial population and soil enzyme activities compared to ConvTill. The micronutrient biofortification (Fe, Zn) in chickpea grains followed the trend of CAc > Cap > ConvTill. The N_2_O emissions remained unaffected under different cropping systems. Interestingly, the CA_c_-management reduced the N_2_O fluxes but with slightly higher CO_2_ emissions, however, curtailing the net GHG-emissions. Triple cropping systems and the CA-management significantly influenced the plant biochemical entities in chickpea viz*.* proline content, super-oxide dismutase, ascorbate peroxidase, catalase and glutathione reductase. Least proline and higher values of superoxide dismutase, glutathione reductase, catalase and ascorbate peroxidase activity in chickpea under CA_c_ indicate the ability of CA-management on moisture-stress tolerance under semi-arid ecologies. Overall, the system-intensification of pearlmillet-chickpea cropping system by the mungbean (PCMCS) coupled with triple zero-tillage and residue-retention (CA_c_) may enhance the chickpea productivity, micronutrient biofortification, moisture-stress tolerance, and soil health with reduced GHG-emissions under prevailing semi-arid conditions of south-Asia. Although, the small-holders still have to maintain a balance while adopting CA_c_ or CA_p_ where livestock rearing equally competes for quality fodder.

## Materials and methods

### Experimental site

**Figure 7:**
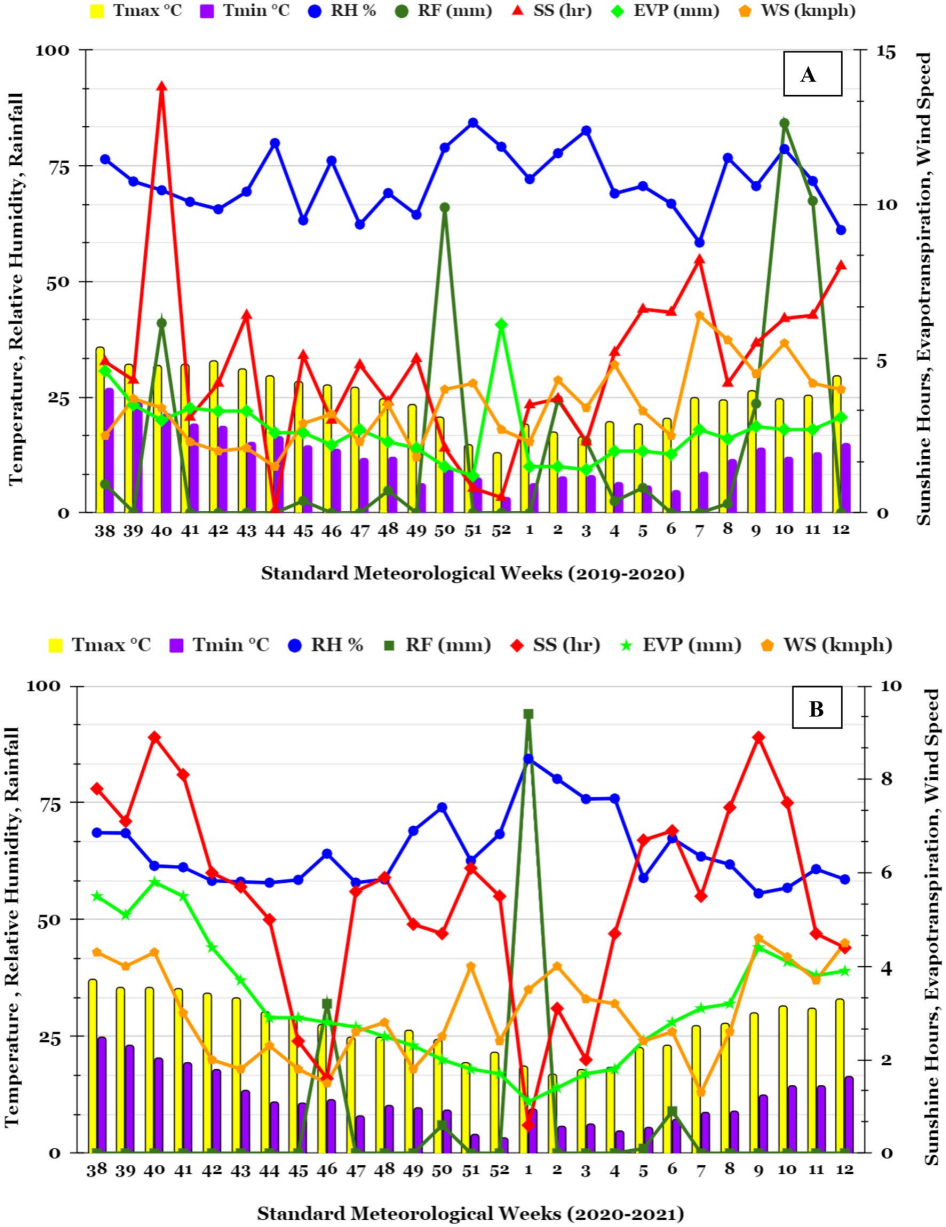
Meteorological data of New Delhi, India for the chickpea growing season (*Rabi* season) corresponding to the year (**A**) 2019–20 and (**B**) 2020–21. *[Note*: Tmax. = Maximum Temperature; Tmin. = Minimum Temperature; RH = Relative humidity; RF = Rainfall; SS = Sunshine hours; EVP = Pan evaporation, WS = Wind speed].

The present experiment was conducted during 2019–2020 and 2020–2021 at research farm of ICAR–Indian Agricultural Research Institute, New Delhi [Latitude 28°4′N; Longitude 77°12′E; Altitude 228.6 m]. The region falls under semi-arid climate having severe winters and hot-dry summers. Almost 70–80% of the annual rainfall (~ 652 mm) is received during July–September and rest 20–30% during October to May^2^. Total rainfall received during the chickpea growing season was 336.7 mm (2019–2020) and 73.7 mm (2020–2021) (Fig. 7). The soil of the experiment was sandy loam in texture (Inceptisol), slightly alkaline in reaction, poor in soil organic carbon (SOC) and available-N and medium in available-P and available-K. Detailed initial physico-chemical properties of experimental soil are enlisted in Table 5.

**Table 5:** Mean physico-chemical properties of soils before initiation of the experiment (0–15 cm soil profile).

S.No	Soil parameters	Value	Methodology used
A	Soil mechanical analysis		Modified hydrometer method^48^
(i)	Sand	61.3%	
(ii)	Silt	12.8%	
(iii)	Clay	25.9%	
(iv)	Textural-class	Sandy loam	USDA soil texture triangle
B	Soil physical analysis		
(i)	Field capacity	18.7%	Pressure plate apparatus^49^
(ii)	Permanent wilting point	6.5%	Pressure membrane apparatus^49^
(iii)	Bulk density	1.51 Mg m^−3^	Core-sampler method^50^
(iv)	Infiltration rate	10.8 mm hr^−1^	Double-ring infiltrometer^51^
C	Soil chemical analysis		
(i)	Organic carbon	0.48%	Walkley and Black method^52^
(ii)	KMnO_4_ oxidizable-N	183 kg ha^−1^	Modified Kjeldahl’s method^53^
(iii)	0.5 N NaHCO_3_ extractable P	16.8 kg ha^−1^	Olsen’s method^54^
(iv)	1 Neutral NH_4_OAc-extractable K	244 kg ha^−1^	Flame photometer method^53^
(v)	pH (1:2.5 soil: water)	7.5	Glass electrode pH meter^49^
(vi)	EC (at 25 °C)	0.31 dS m^−1^	Conductivity bridge^55^

### Treatments detail and crop management

The experiment was laid out in a split-plot design with three replications. In main-plots, tillage and residue management practices were used while diverse cropping systems were allotted in sub-plots (Table 6). Chickpea variety ‘Pusa-1103’ was sown in 2nd week of October during both years, at 45 cm row spacing using 80 kg seed ha^−1^. Gap filling and thinning operations were done within 20 days of sowing. The crop was fertilized with 20 kg N + 40 kg P_2_O_5_ + 40 kg K_2_O per ha. Plant nutrients; N, P and K were applied through urea (46% N), single superphosphate (16% P_2_O_5_) and muriate of potash (60% K_2_O), respectively. The whole amount of NPK fertilizers were applied as basal at sowing of the chickpea. All the crops including chickpea were raised entirely under rainfed conditions on conserved soil moisture. To control weeds, pre-emergence application of pendimethalin was done using 0.75 kg a.i. ha^−1^ in 400 L ha^−1^ spray solution. Under CA plots, after harvest of preceding crop, paraquate 0.75 kg a.i. ha^−1^ was applied using 400 L water ha^−1^ as spray solution. All the crops were grown using standard package of practices except the respective treatment plans^51,56^.

**Table 6:** Description of experimental treatments.

S. no.	Treatment short form	Treatment details
Main-plot (tillage and residue management)		
(i)	Complete CA (CA_c_)	Zero-tillage with crop residue retention @ 3 t ha^−1^ in Crop-I*, 2 t ha^−1^ in Crop-II** on dry-wt. basis and no-residues in Crop-III***. Sowing was done using happy seeder
(ii)	Partial CA (CA_p_)	Zero-tillage without crop residues in any crop. Sowing was done using happy seeder
(iii)	Conventional tillage (ConvTill)	Conventional tillage in all the crops (one deep ploughing using disc plough followed by cultivator thrice and planking)
Sub-plot (Cropping systems)		
(i)	PCCS	Pearl millet–chickpea cropping system
(ii)	PCMCS	Pearlmillet-chickpea–mungbean cropping system
(iii)	PCFCS	Pearlmillet-chickpea–forage pearlmillet cropping system

*Crop-I: Pearlmillet in all the three cropping systems (PCCS, PCMCS, PCFCS); **Crop-II: Chickpea in all the three cropping systems (PCCS, PCMCS, PCFCS); ***Crop-III: Mungbean in PCMCS and forage pearlmillet in PCFCS system.

### Soil sampling and analysis

Current experimentation was done on a long-term experiment. Fresh and moist soil samples from 0 to 15 cm depth were collected immediately after completion of two years of crop rotations of the experiment conducted during 2019–2020 and 2020–2021 i.e. 3rd and 4th year of the long-term experiment. These samples were then transferred to the laboratory for microbial analysis. The total bacterial population was counted using the Pour plating method^57^, in which the samples were incubated on nutrient agar medium for 3 days at 32 °C. Counting of total fungi was performed after incubating the fungal culture plate at 30 °C for 5 days on rose bengal agar medium supplemented with streptomycin (30 µg ml^−1^)^58^. Likewise, total actinomycete counting was done using actinomycete isolation agar (AIA) plates with 50 mg ml^−1^ nalidixic acid^59^, where the AIA plates were incubated for 7 days at 28 °C. The results of triplicate readings were presented as CFU g^−1^ dry soil. The soil acid and alkaline phosphatase enzymatic activities were determined using 16 mM para (*p*)-nitrophenyl phosphate as substrate^60^ and reported as μmol p-nitrophenol g^−1^ h^−1^. Likewise, Glucosidase activity was estimated using 25 mM p-nitrophenol-β-D-glucopyranoside as substrate^61^ and expressed as μmol p-nitrophenol g^−1^ h^−1^. Dehydrogenase activity was determined by the rate of reduction of triphenyltetrazolium chloride to triphenylformazan^51^ and expressed as μg TPF g^−1^ 24 h^−1^. Soil microbial activity expressed as FDA hydrolysis was determined following the method developed by Green et al.^62^.

### Yield parameters and yield estimation

Pods plant^−1^ were counted from 10 randomly selected chickpea plants and there average was taken. Grain yield was recorded (at 14% moisture content) from the net plot area and expressed in t ha^-1^ following the methodology of Rana et al.^51^.

### Plant chemical analysis

Chickpea plant samples (grain and straw) were collected after crop harvest during both the years (2019–20 and 2020–21), and thereafter oven-dried at 60 ± 2 °C for 72 h. Subsequently, samples were grinded in Willey-Mill fitted with stainless steel parts and passed through a 1 mm sieve. Nitrogen concentrations in these samples were estimated by the Micro-Kjeldahl’s method^55^, and total P and K were determined using a sulfuric-nitric-perchloric acid digest^51^. Nutrient uptake was computed by multiplying respective nutrient concentrations by grain and straw yield and expressed in kg ha^-1^. Micronutrient (Fe, Zn) content in grain as well as straw was determined by a di-acid digestion method using atomic absorption spectrophotometry^51^.

### Relative water content

Relative water content (RWC) of the chickpea leaf was determined from first fully expanded top leaf of the plants at flowering stage. Leaf fresh weight was recorded immediately and then the leaf was incubated in distilled water for at least 4 h at 40 °C in the dark, blotted dried and its turgid weight was measured. Finally, dry weight was determined after drying it at 80 °C for 48 h in the oven. The RWC was calculated with the following formula^63^:

1 $$ {\text{RWC }}\left( {\text{\% }} \right) = { }\left( {{ }\frac{{\left( {{\text{F}}_{{\text{w}}} - {\text{D}}_{{\text{w}}} } \right)}}{{\left( {{\text{T}}_{{\text{w}}} - {\text{D}}_{{\text{w}}} } \right)}}{ }} \right){ } \times { }10 $$

Here F_w_ is the fresh weight, D_w_ is the dry weight and T_w_ is the turgid weight.

### Soil moisture content

Soil moisture content were determined at monthly interval during the chickpea crop growing period from 0 to 15 cm soil profile during both the study years i.e. 2019–20, and 2020–21 using standard procedures^51^.

### Plant biochemical properties

Protein, proline content, superoxide dismutase, ascorbate peroxidase, catalase and glutathione reductase activities were determined from chickpea plant samples at flowering stage using standard methodology of Lowry et al.^64^, Bates et al.^65^, Beauchamp and Fridovich^66^, Nakano and Asada^67^, Aebi^68^, and Foyer et al.^69^, respectively.

### GHG-emission studies

Fluxes of greenhouse gases (GHGs) i.e. CO_2_ and N_2_O were measured during both chickpea growing seasons (October to March), using the static chamber method^70,71^ and for continuous 7-days after fertilization and rainfall. Acrylic chambers of 15 cm × 15 cm × 100 cm size, fitted with thermometer, battery operated fan and rubber septa on the top were used for sampling of gases. Samples were collected once in a week between 9 and 11 AM using a 20 ml syringe fitted with a 3-way stopcock, at 0, 30, and 60 min after chamber closure. For each treatment, sampling was carried out in triplicate. CO_2_ and N_2_O concentrations in the collected sample were analyzed by Gas Chromatograph (GC: Hewlett Packard 5890)^70^ having a stainless steel column fitted with a flame ionization and electron capture detector. The cumulative amount of CO_2_ and N_2_O emissions were determined by linear interpolation of two adjacent intervals of measurements carried out on the sampling days assuming that GHGs emissions followed a linear trend during the periods when no sample was taken^72,73^.

The emissions of CO_2_ and N_2_O from soil were calculated by the following equation:

2 $$ {\text{F}} = \rho \times \left( { \frac{{{\text{V}} }}{{\text{A}}}} \right) \times \left( { \frac{{\Delta {\text{c}}}}{{\Delta {\text{t}}}} } \right) \times \left( { \frac{273}{{\text{T}}} } \right) $$

Here F is the CO_2_/N_2_O flux, ρ is the gas density, $V$ is the volume of the close chamber (m^3^), $A$ is the surface area of the closed chamber (m^2^), $\frac{\mathrm{\Delta c}}{\mathrm{\Delta t}}$ represents the rate of increase of CO_2_/N_2_O gas concentration in the chamber (mg/μg m^−3^ h^−1^) and T (absolute temperature) is calculated as 273 + mean temperature (°C) of the chamber. Total CO_2_/N_2_O flux for the entire cultivation period was computed by linear interpolation using the following Eq. ^74^:

3 $$ {\text{Total gas flux}} = \mathop \sum \limits_{i}^{n} \left( {R_{i} \times D_{i} } \right) $$

where ${R}_{i}$ is the CO_2_/N_2_O emission flux (g m^−2^ d^−1^) on the ith sampling interval, ${D}_{i}$ represents the number of days in the ith sampling interval, and *n* is the number of sampling intervals.

### Statistical analysis

The data related to each parameter were analyzed as per the procedure of analysis of variance (ANOVA) to determine treatment effects through Tukey’s honestly significant difference test as a post hoc mean separation test (*p* < 0.05) by using SAS 9.1 software (SAS Institute, Cary, NC). Tukey’s procedure was used where ANOVA was found significant.

### Research involving plants

It is stated that the current experimental research on the plants comply with the relevant institutional, national, and international guidelines and legislation. It is also stated that the appropriate permissions has been taken wherever necessary, for collection of plant specimens. It is also stated that the authors comply with the ‘IUCN Policy Statement on Research Involving Species at Risk of Extinction’ and the ‘Convention on the Trade in Endangered Species of Wild Fauna and Flora’.

## Data Availability

The datasets used and/or analysed during the current study available from the corresponding author on reasonable request.
